# Polymeric Materials for Water and Wastewater Management

**DOI:** 10.3390/polym13010168

**Published:** 2021-01-05

**Authors:** George Z. Kyzas, Athanasios C. Mitropoulos

**Affiliations:** Department of Chemistry, International Hellenic University, 65404 Kavala, Greece

Water is a crucial point of interest nowadays due to its special management. At the same time, wastewater is one of the important pollution types in the water environment. The careful management of water and wastewater is a big challenge and a “hot” trend of recent research. During the last century, a huge amount of wastewater has been discharged into rivers, lakes, and coastal areas. This has resulted in serious pollution problems in aqueous environments. Therefore, it is mandatory to find an appropriate technique in order to efficiently treat and manage water and wastewater. Some indicative/typical methods are biological treatments, adsorption, flocculation, oxidation, membranes, and filtration. All of the above can be achieved by using polymeric materials (polymeric adsorbent materials, polymeric flocculants, polymeric filters, polymeric membranes, polymeric composites, etc.). This Special Issue on “Polymeric Materials for Water and Wastewater Management” sought high-quality works and topics focusing on (but not restricted to) the latest approaches to the management of water and wastewater including biological, chemical, adsorption, flocculation, oxidation, membranes, and filtration using polymeric materials.

This Special Issue on “Polymeric Materials for Water and Wastewater Management”, we believe, succeeded to present such high-quality works and topics focusing on the latest novel wastewater processes using polymeric materials. This Special Issue consists of 11 works (9 research articles, 1 review paper, and 1 teaching note) from distinguished authors worldwide [1,2,3,4,5,6,7,8,9,10,11].

Basheer et al. investigated the use of hybrid carbon nanotubes (CNTs) (prepared by chemical vapor deposition (CVD)) to remove alumina [2], while Iqhrammullah et al. studied the synthesis and characterization of filler-modified castor-oil-based polyurethane foam as an adsorbent material to remove heavy metal ions [5]. Kluczka used, after synthesis, an innovative and environmentally friendly method for boron and manganese removal—from flue gas desulfurization (FGD) wastewater—that is based on a hybrid chitosan-zirconium hydrogel sorbent [6]. The efficient removal of Pb(II) from aqueous solutions by using oil palm biowaste/MWCNT-reinforced polyvinyl alcohol (PVA) hydrogel composites was presented by Zufiqar et al. in the Special Issue, and specifically, that study was focused on kinetics, isotherms, and thermodynamics [11]. In another study, Floros et al. focused on enhancing the flux on hydrophobic polymeric membranes aimed for the direct contact membrane distillation desalination (DCMD) process without compromising salt rejection efficiency [3]. Sun et al. investigated a novel system that was set up by preparing a magnetic flocculant combined with ultraviolet/H_2_O_2_ to realize the rapid enrichment and degradation of diclofenac sodium (DCFS) [7]. Additionally, a composite chitosan/nano-activated carbon (CS-NAC) aminated by (3-aminopropyl)triethoxysilane (APTES) was prepared in the form of beads by Vakili et al. and applied for the removal of acetaminophen from aqueous solutions [8]. Ahualli et al. studied an assembly of soft electrodes and ion exchange membranes for capacitive deionization [1]. Gkika et al. presented an interesting topic of cost estimation of various polymeric adsorbent materials used in literature for wastewater treatment [4], while a review by Yang et al. [10] provides insight into synthesis approaches and structural properties of recent reverse osmosis (RO) and nanofiltration (NF) membranes which are used to retain dissolved species such as heavy metals, electrolytes, and inorganic salts in various aqueous solutions. A specific focus of that article was placed on introducing and comparing the water purification performances of different classes of polymeric and ceramic membranes in related water-treatment industries. A technical report by Xia et al. demonstrated a strategy to design a modified starch/polyvinyl alcohol composite (CCSP) which was employed as a highly efficient and economical fixed-bed adsorbent for treating textile wastewater [9].

Many authors, whom we—as editors—thank very much, from various countries contributed marvellously to the present Special Issue. All the aforementioned topics and many more were explored in detail. Certainly, the field of wastewater treatment using polymeric materials and its processes is vast; the present study hopefully adds one more useful contribution.
